# Anthropometric Characterization and Physical Performance by Age and Biological Maturation in Young Tennis Players

**DOI:** 10.3390/ijerph182010893

**Published:** 2021-10-17

**Authors:** Pablo Luna-Villouta, Marcelo Paredes-Arias, Carol Flores-Rivera, Claudio Hernández-Mosqueira, Ricardo Souza de Carvalho, César Faúndez-Casanova, Jaime Vásquez-Gómez, Rodrigo Vargas-Vitoria

**Affiliations:** 1 Facultad de Ciencias de la Educación, Pedagogía en Educación Física, Universidad San Sebastián, Concepción 4030000, Chile; pablo.luna@uss.cl (P.L.-V.); marcelo041192@gmail.com (M.P.-A.); 2 Facultad de Ciencias de la Educación, Universidad Católica del Maule, Doctorado en Ciencias de la Actividad Física, Talca 3460000, Chile; 3 Facultad de Educación y Ciencias Sociales, Universidad Andrés Bello, Concepción 4030000, Chile; carolfloresrivera@gmail.com; 4 Departamento de Educación Física, Deportes y Recreación, Universidad de La Frontera, Temuco 4780000, Chile; claudiomarcelo.hernandez@ufrontera.cl; 5 Facultad de Educación, Pedagogía en Educación Física, Universidad Católica del Maule, Talca 3460000, Chile; rsouza@ucm.cl (R.S.d.C.); cfaundez@ucm.cl (C.F.-C.); 6 Centro de Investigación de Estudios Avanzados del Maule (CIEAM), Universidad Católica del Maule, Talca 3460000, Chile; jvasquez@ucm.cl; 7 Laboratorio de Rendimiento Humano, Universidad Católica del Maule, Talca 3460000, Chile

**Keywords:** maturation, physical performance, anthropometry, tennis, young boys

## Abstract

The objective was to analyze anthropometric and physical performance variables as a function of chronological age and biological maturity in young Chilean tennis players. The study was observational, cross-sectional, with descriptive and analytical characteristics. Eighty-seven tennis players were evaluated (58 men 15.1 ± 0.8 years and 29 women, 15.3 ± 0.8 years). The measured anthropometric variables were a sprint test of 20m; a modified agility test; a sit-and-reach test and shoulder flexibility; manual grip strength; horizontal jump in feet; a medicine ball throw; a countermovement vertical jump; an abalakov vertical jump and a 20-m shuttle-run test. The growth velocity acceleration peak (APHV), skeletal muscle mass and fat mass were calculated, R^2^ and standard error of estimate (SEE) were examined. The results show that chronological age explained the anthropometric variables between 1 and 23% in men and 1 and 29% in women; by biological age, variables were explained between 3 and 53% in men and 2 and 42% in women. Of the physical performance variables, chronological age described between 2 and 24% of them in men and 1 and 29% in women; the same were explained by biological age between 1 and 19% in men and 1 and 26% in women. We conclude that anthropometric variables showed a better relationship with biological age, except for volume of fat tissue, while physical performance variables showed low association with both biological and chronological age.

## 1. Introduction

In recent years, like other sports, tennis has changed from an eminently technical sport, with specific technical skills as predominant factors (for example, skills in handling the racket and control of the ball) to a more dynamic and explosive sport characterized by a greater hitting power and speed, which is remarkably physically demanding as compared with historical play styles [1]. 

In this current scenario, tennis players require high performance in most of the components associated with physical fitness, such as speed, agility, strength, and power, among others [2,3]. Thus, in both professional and youth tennis, physical condition is considered essential forachieving high performance, thus this assessment must be developed [4,5,6].

The evaluation of different parameters associated with sports performance can provide coaches and players with important information for decision-making and the planning of training and competition [7]. This may also be essential for the selection of young talent [8,9], for the prevention of injuries due to overtraining [10] and to maintain the motivation [11].

Morphological characteristics, body composition, and physical qualities vary depending on growth, maturation, and external factors such as nutrition and physical effort, among others [4]. Adolescence is defined as a period of rapid and significant change in morphological and physiological attributes that can affect physical performance [12]. In addition, young people with advanced stages of maturity usually show better results in motor, physical and functional evaluations compared with their peers in chronological age [13]. Although the functional differences associated with maturity are, to a certain extent, transitory, the differences among the young people of contrasting maturity are reduced and even usually disappear, later, when late-maturing athletes reach higher levels of maturation at the end of adolescence or in the beginning of adulthood [12,14,15].

This effect during adolescence can be advantageous, in terms of gains in body size, strength and muscle power; but it can also negatively affect aspects such as body composition; as an example, the increase in fat tissue, in women. Another effect is observed in coordination and agility due to the rapid increase in body size and weight in men and women [14]. This knowledge of the level of maturation development, body size, physical performance and the relationship between the different physical attributes associated with sports performance in tennis (speed, power, agility, flexibility, muscular strength of the upper and lower extremities), could help determine the relative importance of such measures. In addition, it may provide essential inputs for the optimal development of training programs aiming to improve athletic performance. [4,16].

In this sense, scientific evidence indicates that it is important to address in a more satisfactory way the athletic potential of young tennis players, taking as a reference their maturity levels [9,12,17]. There are still limitations in the study of the needs and components associated with the performance of adolescent tennis players [18]. The regular monitoring of biological maturity is recommended to allow training programs to be designed with an appreciation for the physical and physiological processes that occur because of maturation [3]. It has been observed that biological maturation should be monitored to optimize adaptation to training and minimize the risk of activity-related injuries [19], to distinguish whether maturation or exposure to regular exercise is responsible for the observed changes in performance [20].

Up to date, several studies, mainly from the northern hemisphere, have addressed the performance needs of adolescent competitive tennis players, indicating that more information is needed about the role of maturity and growth in physical and athletic performance [1,9,10,14,17,21,22,23,24]. However, in South America, such studies seem to be still incipient, focusing mainly on the study of anthropometric aspects [25,26] and the external load of the game [27], without considering its associations with levels of biological maturation.

The foregoing shows that, in general terms, regarding the data, the study of the growth and physical performance capacities of youth tennis players in the context of biological maturity is still limited. This, considering that chronological age is not useful for monitoring growth, maturation, and physical performance [12,14,15]. Furthermore, maturation and growth are influenced by external factors (such as nutrition, family environment and physical exercise) and internal factors (such as race and sex), which act independently or together on the potential genetic status of the individual [15,20]. Therefore, the study these variables can have multiple benefits for sports performance and in the development of training programs.

Based on these assumptions, the objective of the study was to analyze the anthropometric and physical performance variables as a function of chronological age and biological maturity in young elite Chilean tennis players.

## 2. Materials and Methods

An observational cross-sectional study, with descriptive and analytical characteristics, in which 87 Chilean youth tennis players of both sexes voluntarily participated, who were selected through a non-probabilistic convenience sampling, was conducted. The sample size was estimated using the statistical software G * Power v. 3.1.9.7 (Heinrich-Heine-Universität Düsseldorf, Düsseldorf, Germany); a priori analysis for the difference of means (unpaired t-test), with a bilateral alpha error of 5%, an effect size 0.75 and a statistical power of 80%, which marked a minimum sample of 29 subjects by sex. The inclusion criteria were the following: (1) being a Chilean competitive tennis player between 14 and 16 years old; (2) training systematically, with a minimum weekly volume of 10 h in the last 12 months; (3) having participated in international tournaments in the last two years. The exclusion criteria were: (1) a failure to complete all the evaluations; (2) a failure to show up with appropriate clothing or sports shoes for physical evaluations; (3) presenting with a physical injury that would prevent maximum performance or affect the result of the evaluations. For the data collection process, authorization was requested from the directors of the different tennis clubs, by means of an invitation letter and with detailed information about the objectives and procedures of the study. Once clubs had accepted, informed consent forms were sent to the subjects’ parents, giving an account of the objective of the research and the evaluation procedures. Finally, the tennis players signed the agreement, confirming their voluntary participation in the evaluations. The ethics committee of the Universidad San Sebastián, Chile (protocol number USS 51-2018-20) approved the study.

### 2.1. Procedures

The anthropometric evaluations were carried out in the morning, before any type of exercise, in a private and specially equipped room, which allowed individual measurements to be taken. A trained assessor, following the standard procedures of Marfell-Jones et al. (2012) [28], carried out the measurements. Body weight (kg) was measured using a mechanical scale (Seca 700, Hamburg, Germany), with a precision of 50 grams, ranging from 0 to 220 kg. Height (cm) was measured according to the Frankfurt plane without shoes, using an aluminum stadiometer of Seca 220 brand (Hamburg, Germany), graduated in millimeters; its scale was (0.60–2.20 mm). The sitting height (trunk–head height) was taken using a wooden bench with a height of 0.50 m, with a measurement scale of 0 to 1.50 m and with a precision of 1 mm. In the case of skin folds (triceps brachial, anterior thigh and medial leg), measurement was done with a Harpenden Skinfold^®^ (Baty International Ltd, West Sussex, UK) anthropometric forceps. The perimeters of the arm, thigh, and leg were measured with a Lufkin^®^ (Medina, OH, United States) Metallic anthropometric tape. All anthropometric variables were measured three times. The technical measurement error ranged from 0.15% to 0.9%.

The percentage of body fat was calculated with the regression equations of Slaughter et al. (1988) [29] and muscle mass was obtained using the equation for adolescents proposed by Poortmans et al. (2005) [30] Biological maturation was determined by years of peak growth-rate acceleration (APHV), proposed by Mirwald et al. (2002) [31], and it was predicted by regression equations for both men and women.

The physical performance tests were carried out in the morning period, after the anthropometric ones, on tennis courts, on a clay surface. Those evaluated had to wear shorts, athletic shirt, and sports shoes, to match competition clothing. Two experienced evaluators (holding Masters in Sports Sciences, MSc.) were in charge of the evaluations. They were also trained to administer the tests in learning and practice sessions. Prior familiarization sessions were held for the participants in which they received instructions and explanations from the researchers as to the correct performance of the tests. All those evaluated were able to undergo the tests, performing them between three and five times, in the week prior to carrying out the evaluations, except for the 20 m shuttle run test, originally proposed by Léger et al. (1988). On the day of data collection, the tests were executed twice by each subject, where the best values were recorded, except for the 20 m shuttle run test, which was performed only once. The breaks between each test were 3 to 5 min long, and carried out according to the following protocol: a 15-min warm-up was performed, with general physical exercises and stretching. The application sequence was: first, the speed tests (sprint 20 m), wherein the participant had to be in a high starting position behind the starting line, and, at the signal, would travel the distance in the shortest time possible. Second, the modified agility test (MAT) was carried out. This test involves moving and changing direction over a total distance of 20 m at maximum speed. For this test, four cones were arranged in the shape of a “T”, the subject sprinted in a straight line to the first cone placed at 5 m, and then to a second cone placed 2.5 m to his left. This was done by moving laterally without crossing the feet. The subject then moves in the same manner to the right side to reach the third cone, placed at 5 m. They then return to the middle cone and finish at the starting position. The drill was considered to have been completed correctly when the base of the cone was touched. Both tests were recorded by means of a manual digital stopwatch. Third, the flexibility assessment, sit and reach (forward trunk flexion), was performed using a millimeter drawer. Fourth, the shoulder flexibility test, using a millimeter wooden cane, was performed. These were followed by the evaluation of manual grip strength (MGS), which was carried out using a Jamar Sammons Preston manual hydraulic dynamometer (kg), in the dominant hand and with the subject standing. Following this, the horizontal jump, with feet together (HJ) was performed; then, a medicine ball throw (MBT) with both hands over the head was performed, for which a 3 kg ball was used. To measure the results of both tests, a Stanley Power Lock millimeter tape was used. Subsequently, vertical jumps (VJ) were executed, which were recorded with the Globus Ergo Jump platform (Bosco System), as follows: first the countermovement jump test (VJ CMJ) and then the abalakov (VJ ABK) were performed, according to the recommendations of Bosco and Padulles [32]. Finally, for the evaluation of aerobic performance, a 20-m shuttle run test or the Course–Navette test were used, by means of a round trip at the rhythm indicated by a auditory signal (CD-ROM with an auditory signal) and following the recommendations of Léger et al. (1988) [33], the total distance covered by each tennis player was considered as the result.

### 2.2. Statistical Analysis

Statistical analysis was carried out using GraphPad Prism software 7.0 (San Diego, CA, United States) and 17.0 version of the SPSS IBM Corp. (Somers, NY, United States) statistical package. The data obtained are presented as the descriptive statistics of mean, standard deviation (SD), median, first and third quartiles, 95% confidence interval (CI), lower limit (LL), upper limit (UL) and coefficient of variation (CV). The Kolmogorov–Smirnov test was used to determine the normal distribution of the variables, which followed a normal distribution. The relationship between the variables was verified through Pearson’s correlation coefficient. Simple regression analysis was carried out, where the R^2^ and standard error of estimate (SEE) were analyzed for both chronological age and maturational age (APHV) and the anthropometric and physical performance variables were analyzed by sex. The differences between men and women were determined by means of the t-test for independent samples. The level of significance used for all study variables was *p* < 0.05.

## 3. Results

In Table 1 the descriptive data are expressed as mean, standard deviation (SD), minimum (Min), maximum (Max), median, first and third quartile values, 95% confidence interval (CI 95%), lower limit (LL), upper limit (UL) and coefficient of variance (CV).

**Figure 1:**
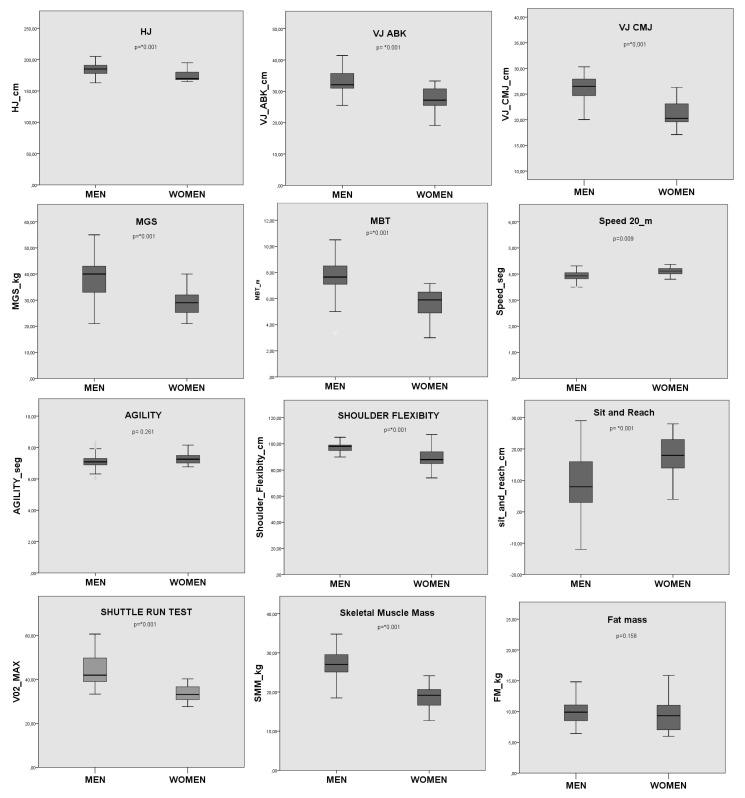
Comparison of mean and standard deviation in measures of physical performance and body composition (*: significant difference).

When comparing the anthropometric variables significant differences were observed between genders: men present higher values of body weight (M = 64.3 ± 7.9 kg; W = 50.1 ± 6.7 kg. *p* = 0.001); height (M = 174.2 ± 7.6; W = 164.9 ± 3.7, *p* = 0.001). Regarding body composition, significantly higher values were observed in men for skeletal muscle mass (M = 26.9 ± 4.1; W = 19.0 ± 2.8, *p* = 0.001), while the body fat did not show significant differences between males and females. On the other hand, in the physical performance variables, there were significant differences; men showed better results than women in HJ (M = 185.5 ± 10.5 cm; W = 173.6 ± 7.4 cm, *p* = 0.001), VJ CMJ (M = 25.8 ± 3.8 cm; W = 20.9 ± 3.6 cm, *p* = 0.001), VJ ABK (M = 32.7 ± 4.2 cm; W = 27.4 ± 4.1 cm, *p* = 0.001), FPM (H = 39.4 ± 7.0 kg; M = 29.2 ± 5.1 kg, *p* = 0.001), MGS (M = 7.7 ± 1.3 m; W = 5.8 ± 1.1 m, *p* = 0.001) and 20-m shuttle run test (M = 2654.1 ± 660.3 m; W = 1765.5 ± 302.4 m, *p* = 0.001). For their part, women showed better results in the sit and reach flexibility tests (M = 9.1 ± 10.2 cm; W = 18.3 ± 5.8, *p* = 0.001) and flexibility of shoulders tests (M = 97.8 ± 5.6 cm; W = 87.6 ± 8.5 cm, *p* = 0.001). No significant differences were observed in the 20 m speed and agility tests. These results are shown in Figure 1.

The values for the simple linear regression for the chronological and biological age are shown in Table 2. It is observed that biological age (APHV) is better associated compared with chronological age in the anthropometric variables of height (M = 53%; W = 42%), body weight (M= 29%; W= 30%) and skeletal muscle mass (SMM) (M= 60%; W = 32%). For fat mass, both chronological age (M = 1%; W = 1%) and biological age (M = 17%; W = 2%) showed very low influence on both sexes.

Regarding the physical performance variables (Table 2), the explanation percentages are very low for both chronological age and biological age (APHV). The highest values for chronological age are found in the tests of MBT (M= 24%; W= 29%), agility (M = 19% and W = 23%) and MGS (M = 18%; W = 15%). For biological age (APHV), the best values were found in the tests of agility (M = 19%; W = 26%), MBT (M = 17%; W = 24%) and MGS (M = 13%; W = 10%).

## 4. Discussion

The objective of this study was to analyze the anthropometric and physical performance variables based on chronological age and biological maturity in young elite Chilean tennis players. The results show that for the anthropometric variables of height, weight and SMM, there is a better relationship with biological age than for chronological age, except for fat tissue, which showed very low influence for both ages.

The high explanatory values of the APHV demonstrate the convenience of using this type of reference in young athletes, which has been reported by other studies, both in youth tennis players [14] and in other sports [34]. It has been reported [9] that, when monitoring anthropometric characteristics and maturation, there are significant differences between tennis players of the same chronological age. These differences, especially anthropometric ones, tend to influence the style of play, giving taller players greater speed and range of shots [1]. Specifically, height in tennis is a determining element for the execution of strokes, especially, for the serve, which, if well placed and powerful, is fundamental, from a strategic point of view, for the development of the game, even at the junior level [8]. In addition, somatic maturation appears to be a better predictor of body modifications than chronological age. Biological maturation plays a determining role in increasing skeletal muscle mass and muscle strength [15], which highlights the importance of controlling this type of anthropometric parameter with this technique, to achieve adequate monitoring of the training effects, competition, and injury prevention [12]. A greater muscular component has been related to a higher level of training and physical performance, the development of an improved stroke and fast and powerful tennis movements [35]. Furthermore, the physical demands of tennis training become greater as youth tennis players participate in more important competitions in junior or in adult tournaments, increasing both the intensity and the duration of the games, such that more muscle power is necessary [36]

The findings of low explanatory percentages with respect to body fat, for chronological age and biological age in both genders are contrary to those reported in other studies, which found more significant relationships between these indicators [1,17]. These studies included European tennis players and higher age ranges in their subject pools, which may explain these differences. It has been reported [20] that the measurements are less precise when extending beyond the APHV period. In addition, homogeneous groups, such as the tennis players evaluated in this study, may account for the absence of significant differences in maturation. Another aspect that may explain the differences in the reviewed studies [1,17] is the level of training of the groups, which produces body and organic adaptations due to the volume and intensity of exercise. Additionally, fat tissue and body composition are subject to the influence of various external and internal factors, such as the level of physical exercise, age, maturation, nutrition, socioeconomic status, genetic inheritance and physical environment, among others [37]. Along with the above, it is necessary to consider that biological maturation in relation to intensity and duration is different for each subject, regardless of age, race or sex.

On the other hand, with respect to the physical performance variables, the association percentages were very low for both chronological age and biological age; these values are lower than those reported by other studies in young tennis players [10,14,21]. However, the values found in MBT, MGS, and agility in chronological age (between 15 to 29%), and biological age (between 10 to 26%) for both sexes are like those found by other investigations in young tennis players [1,17]. These results show a relative value for biological maturation in physical performance tests related to this sport, such as muscular strength, power, and agility. It has been reported [1,9] that biological maturation is not always accompanied by increased physical performance. This difference with our results can be explained by the idea that the interaction between genetics and the environment is complex and not additive [38]. Therefore, maturation depends on various internal and external factors, as previously described. Along these lines, it seems that the best correlations and influences of maturation with physical performance are found in young tennis players between 11 and 13 years of age, in whom these effects seem to decrease from 15 to 16 [23], and these ages correspond to those evaluated in this study (men = 15.4 ± 0.8 years; women = 15.3 ± 0.8 years). The differences in physical and functional performance associated with maturity are raised, to some extent, as transitory, since the maturity differences between young people are reduced, and in some cases, eliminated in late adolescence or adulthood. Another factor that may explain our differences with previous studies [1,9,10,14,21] is that they assessed European tennis players, a population with differences from Chilean tennis players, for example, in race and other environmental factors (socioeconomic level and nutrition), which we have already described as intervening factors, and which were not controlled for. Furthermore, it has been reported that specific physical preparation methodologies directly influence the physical performance of young tennis players, and that maturation can alter training objectives [39]. Meanwhile, it should be noted that intensive physical training does not negatively affect somatic growth in sports that do not require strict dietary restrictions, which leads to an energy imbalance [40]. This information, together with the results obtained, show that it is still difficult to generalize the results and analysis from the general population of adolescents to elite young athletes, even more so in a specific sport or between different sports disciplines [14].

It should be noted that other studies whose results focused on physical performance, and which were found to be contrasting [1,10,14,17,21,23] used different techniques and indicators of biological maturation, such as the Tanner scale and radiographs, among others. In this sense, the APHV presents, as its main advantages, the use of non-invasive techniques, such as anthropometry, presenting itself as a simple tool of practical use and low operational cost compared with other biological indicators [38].

This study presents limitations in the selection and numerosity of the evaluated sample. Those evaluated were very similar in their level of training and competence, which restricts their transfer to other levels. In addition, cross-sectional data were collected, which may limit the explanatory inferences in relation to the findings. Therefore, the results obtained should be used as a standard reference but interpreted with caution in youth tennis players. The main strength of this study is that it addresses a topic that is not explored in tennis in South America, and that is considered essential for young athlete’s preparation. Along with this, APHV is used, is a non-invasive, simple, and fast technique, administrable in different populations. For these reasons, the results of this study may have implications for strengthening the process of identification and sports training of young Chilean tennis players by means of maturational, anthropometric and physical performance information necessary for the preparation of their respective short-, medium-, and long-term training programs.

## 5. Conclusions

Based on the results obtained, we conclude that anthropometric variables show better a relationship with biological age than with chronological age, except for fat tissue, which showed a very low influence by the evaluated ages. The physical performance variables showed low association for both biological and chronological age. The study, in turn, shows significant differences between men and women, in the anthropometric variables of height, body weight and SMM. Furthermore, better results were observed in the physical performance tests for men in the HJ, VJ CMJ, VJ ABK, MGS, MBT and 20-m shuttle run test, except for the flexibility tests, which favored women.

## Figures and Tables

**Table 1:** Descriptive Characterization of the Sample.

Variables	Men (*n* = 58)										Women (*n* = 29)									
	Mean	ST	Min	C25	Median	C75	Max	CI 95%		CV (%)	Mean	ST	Min	C25	Median	C75	Max	CI 95%		CV (%)
								LL	UL									LL	UL	
Age (years)	15.4	0.8	14.0	14.9	15.5	16.1	16.6	15.2	15.6	4.9	15.3	0.8	14.0	14.8	15.2	15.9	16.7	15.0	15.6	4.9
APHV (levels)	0.4	1.0	−2.1	−0.4	0.6	1.1	2.2	0.1	0.7	242.4	4.2	0.9	2.6	3.6	4.3	4.9	5.9	3.9	4.6	21.4
Height (cm)	174.2	7.6	153.0	171.0	175.5	179.0	196.0	172.2	176.2	4.4	164.9	3.7	157.0	163.5	165.0	168.0	169.0	163.5	166.3	2.2
BW (kg)	64.3	7.9	42.7	60.3	65.1	69.6	80.7	62.3	66.4	12.3	50.1	6.7	40.5	44.9	49.3	55.8	67.0	47.5	52.6	13.5
FM (kg)	10.7	3.4	6.4	8.6	10.0	11.9	23.0	9.8	11.5	31.5	9.6	3.2	6.0	7.0	9.4	11.3	20.3	8.4	10.8	33.4
SMM (kg)	26.9	4.1	16.6	25.0	27.0	29.7	39.3	25.8	28.0	15.4	19.0	2.8	12.7	16.5	19.2	20.9	24.2	17.9	20.1	14.7
PBF (%)	16.5	4.1	10.0	13.5	15.6	18.2	29.6	15.4	17.6	24.8	18.8	4.6	12.7	14.9	18.8	22.4	30.3	17.1	20.6	24.4
SMM (%)	42.1	6.3	27.3	38.4	41.7	46.0	63.3	40.5	43.8	14.8	38.0	6.0	27.2	34.2	36.8	44.0	47.3	35.8	40.3	15.8
Training hours / week	15.0	4.5	10.0	10.5	15.0	20.0	20.0	13.5	15.4	30.2	13.6	4.0	10.0	10.0	10.5	17.5	20.0	11.6	14.8	29.8
HJ (cm)	185.5	10.5	163.0	177.8	185.0	191.3	215.0	182.7	188.2	5.7	173.6	7.4	165.0	168.0	170.0	180.0	195.0	170.8	176.4	4.3
VJ CMJ (cm)	25.8	3.8	16.3	24.5	26.5	27.9	37.5	24.8	26.8	14.9	20.9	3.6	12.6	19.5	20.3	23.2	30.1	19.5	22.3	17.1
VJ ABK (cm)	32.7	4.2	20.1	30.9	32.1	35.8	41.4	31.6	33.8	12.7	27.4	4.1	14.7	25.4	27.2	30.9	33.3	25.9	29.0	15.0
MGS (kg)	39.4	7.0	21.0	33.0	40.0	43.3	55.0	37.5	41.2	17.9	29.2	5.1	21.0	25.2	29.0	32.0	40.0	27.2	31.1	17.6
MTB (mts)	7.7	1.3	3.4	7.1	7.7	8.5	10.5	7.3	8.0	16.9	5.8	1.1	3.0	4.8	5.9	6.5	7.2	5.3	6.2	18.3
Speed 20 m (s)	3.9	0.3	3.0	3.8	3.9	4.1	5.0	3.8	4.0	7.7	4.1	0.3	3.0	4.0	4.1	4.2	4.5	4.0	4.2	6.6
Agility (s)	7.2	0.6	6.0	6.9	7.1	7.3	8.9	7.0	7.3	8.0	7.3	0.3	6.8	7.0	7.3	7.5	8.2	7.2	7.4	4.6
Sit and reach (cm)	9.1	10.2	−12.0	2.8	8.0	16.3	29.0	6.4	11.8	112.0	18.3	5.8	4.0	13.5	18.0	23.5	28.0	16.1	20.6	31.9
Shoulder flexibility (cm)	97.8	5.6	87.0	95.0	98.0	99.3	120.0	96.3	99.3	5.7	87.6	8.5	66.0	84.0	88.0	94.0	107.0	84.4	90.8	9.7
Shuttle run test (m)	2654	660	1540	2115	2550	3240	3860	2481	2828	25	1766	302	1200	1480	1740	2010	2420	1650	1881	17.1

Note: BW—body weight; APHV—peak growth rate acceleration; FM—fat mass; SMM—skeletal muscle mass; PBF—body fat percentage; HJ—horizontal jump; VJ CMJ—vertical jump with countermovement; VJ ABK—vertical jump abalakov; MGS—manual grip strength; MBT—medicine ball throw.

**Table 2:** Simple linear regression values between chronological age and biological age with anthropometric and physical performance variables.

Variables	Men (*n* = 58)								Women (*n* = 29)							
	Chronological Age (Years)				Biological Age (APHV)				Chronological Age (Years)				Biological Age (APHV)			
	R	R^2^	SEE	*p*	R	R^2^	SEE	*p*	R	R^2^	SEE	*p*	R	R^2^	SEE	*p*
Height (m)	0.48	0.23	6.78	0.001	0.73	0.53	5.28	0.001	0.54	0.29	3.13	0.002	0.65	0.42	2.82	0.001
BW (kg)	0.41	0.17	7.26	0.001	0.54	0.29	6.69	0.001	0.35	0.12	6.43	0.005	0.55	0.30	5.73	0.002
FM (kg)	0.08	0.01	3.38	0.905	0.17	0.03	3.34	0.202	0.09	0.01	3.25	0.682	0.14	0.02	3.23	0.460
SMM (kg)	0.35	0.12	3.93	0.008	0.60	0.36	3.34	0.001	0.43	0.18	2.58	0.022	0.56	0.32	2.35	0.001
HJ (cm)	0.15	0.02	10.50	0.249	0.13	0.02	10.54	0.331	0.08	0.01	7.53	0.841	0.12	0.02	7.48	0.522
VJ CMJ (cm)	0.29	0.08	3.72	0.029	0.30	0.09	3.70	0.023	0.08	0.01	3.64	0.858	0.08	0.01	3.64	0.918
VJ ABK (cm)	0.26	0.07	4.06	0.052	0.32	0.10	3.98	0.001	0.08	0.01	4.17	0.749	0.08	0.01	4.18	0.836
MGS (kg)	0.42	0.18	6.44	0.001	0.36	0.13	6.62	0.005	0.38	0.15	4.83	0.032	0.31	0.10	4.97	0.005
MBT (mts)	0.49	0.24	1.14	0.001	0.41	0.17	1.19	0.002	0.54	0.29	0.91	0.003	0.49	0.24	0.93	0.007
Speed 20 m (s)	0.04	0.00	0.30	0.753	0.08	0.01	0.30	0.744	0.16	0.03	0.27	0.403	0.09	0.01	0.27	0.633
Agility (s)	0.43	0.19	0.52	0.001	0.44	0.19	0.52	0.001	0.48	0.23	0.30	0.005	0.51	0.26	0.29	0.005
Sit and reach (cm)	0.06	0.00	10.25	0.677	0.08	0.01	10.25	0.693	0.16	0.02	5.88	0.419	0.12	0.02	5.91	0.526
Shoulder flexibility (cm)	0.19	0.04	5.53	0.143	0.08	0.01	5.64	0.856	0.38	0.14	8.01	0.045	0.30	0.09	8.26	0.119
Shuttle run test (m)	0.26	0.07	643.05	0.047	0.08	0.01	665.77	0.788	0.30	0.09	293.39	0.109	0.40	0.16	282.85	0.034

Note: BW—body Weight; APHV—peak growth rate acceleration; FM—fat mass; SMM—skeletal muscle mass; HJ—horizontal jump; VJ CMJ—vertical jump with countermovement; VJ ABK—vertical jump abalakov; MGS—manual grip strength; MBT—medicine ball throw.

## Data Availability

The data presented in this study are available on request from the corresponding author. The data are not publicly available due to privacy and anonymity.
